# Supplementary material for: Learning nonlinear operators in latent spaces for real-time predictions of complex dynamics in physical systems
Source: Nat Commun. 2024 Jun 14;15:5101. doi: 10.1038/s41467-024-49411-w (PMC11178876; doi:10.1038/s41467-024-49411-w)
Supplement: Supplementary file 1 — Supplementary Information [file 41467_2024_49411_MOESM1_ESM.pdf]

Supplementary Information

Learning nonlinear operators in latent spaces for real-time predictions of complex dynamics in physical systems

Nomenclature

Supplementary Table 1. Summary of the main symbols and notation used in this work.

Notation	Description
$\mathbf{x}_j$	an input realization (e.g., ICs, BCs)
$\mathbf{y}_j$	an output of the PDE model
$f(\cdot)$	a forcing function of the PDE
$\mathscr{G}$	PDE solution operator
$\mathscr{G}_\theta$	approximation of mapping on latent space
θ	trainable parameters of the neural operator
$\mathscr{I}_{\theta_{\text{encoder}}}$	encoder part of the autoencoder
$\mathscr{I}_{\theta_{\text{decoder}}}$	decoder part of the autoencoder
$\{x_i\}_{i=1}^m$	sensor locations
m_s, m_t	spatial and temporal discretization
$[\mathbf{x}_j^r(x_1), \mathbf{x}_j^r(x_2), ..\mathbf{x}_j^r(x_d)]$	pointwise evaluation of the reduced input to the branch net
ζ	locations as inputs to the trunk net
$\mathscr{L}_{\text{ae}}$	autoencoder loss
$\mathscr{L}_r(\boldsymbol{\theta})$	L-DeepONet residual loss
d	latent space dimensionality
GRF	Gaussian random field
CNN	convolutional neural network
FNN	feed-forward neural network
CAE	convolutional autoencoder
VAE	variational autoencoder
MLAE	multi-layer autoencoder
N	total number of train/test data
OOD	out-of-distribution
KLE	Karhunen-Loéve expansion
MSE	mean squared error

Theoretical details

Neural operators

Let $\Omega \subset \mathbb{R}^D$ be a bounded open set and $\mathscr{X} = \mathscr{X}(\Omega;\mathbb{R}^{d_x})$ and $\mathscr{Y} = \mathscr{Y}(\Omega;\mathbb{R}^{d_y})$ two separable Banach spaces. Furthermore, assume that $\mathscr{G} : \mathscr{X} \to \mathscr{Y}$ is a non-linear map arising from the solution of a time-dependent PDE. The objective is to approximate the nonlinear operator via the following parametric mapping

$$\mathscr{G} : \mathscr{X} \times \Theta \to \mathscr{Y} \quad \text{or,} \quad \mathscr{G}_\theta : \mathscr{X} \to \mathscr{Y}, \ \ \theta \in \Theta \tag{1}$$

where Θ is a finite dimensional parameter space. The optimal parameters θ^* are learned via the training of a neural operator with backpropagation based on a dataset $\{\mathbf{x}_j, \mathbf{y}_j\}_{j=1}^N$ generated on a discretized domain $\Omega_m = \{x_1, \dots, x_m\} \subset \Omega$ where $\{x_j\}_{j=1}^m$ represent the sensor locations, thus $\mathbf{x}_{j|\Omega_m} \in \mathbb{R}^{D_x}$ and $\mathbf{y}_{j|\Omega_m} \in \mathbb{R}^{D_y}$ where $D_x = d_x \times m$ and $D_y = d_y \times m$.

DeepONet

The Deep Operator Network (DeepONet)[5] aims to learn operators between infinite-dimensional Banach spaces. Learning is performed in a general setting in the sense that the sensor locations $\{x_i\}_{i=1}^m$ at which the input functions are evaluated need

not be equispaced, however they need to be consistent across all input function evaluations. Instead of blindly concatenating the input data (input functions $[\mathbf{x}(x_1), \mathbf{x}(x_2), \ldots, \mathbf{x}(x_m)]^T$ and locations ζ) as one input, i.e., $[\mathbf{x}(x_1), \mathbf{x}(x_2), \ldots, \mathbf{x}(x_m), \zeta]^T$, DeepONet employs two subnetworks and treats the two inputs equally. Thus, DeepONet can be applied for high-dimensional problems, where the dimension of $\mathbf{x}(x_i)$ and ζ no longer match since the latter is a vector of d components in total. A trunk network $\mathbf{f}(\cdot)$, takes as input ζ and outputs $[tr_1, tr_2, \ldots, tr_p]^T \in \mathbb{R}^p$ while a second network, the branch net $\mathbf{g}(\cdot)$, takes as input $[\mathbf{x}(x_1), \mathbf{x}(x_2), \ldots, \mathbf{x}(x_m)]^T$ and outputs $[b_1, b_2, \ldots, b_p]^T \in \mathbb{R}^p$. Both subnetwork outputs are merged through a dot product to generate the quantity of interest. A bias $b_0 \in \mathbb{R}$ is added in the last stage to increase expressivity, i.e., $\mathscr{G}(\mathbf{x})(\zeta) \approx \sum_{i=k}^{p} b_k t_k + b_0$. The generalized universal approximation theorem for operators, inspired by the original theorem introduced by[1], is presented below. The generalized theorem essentially replaces shallow networks used for the branch and trunk net in the original work with deep neural networks to gain expressivity.

Theorem 1 (Generalized Universal Approximation Theorem for Operators.) *Suppose that X is a Banach space, $K_1 \subset X$, $K_2 \subset \mathbb{R}^d$ are two compact sets in X and $\mathbb{R}^d$, respectively, V is a compact set in $C(K_1)$. Assume that: $\mathscr{G} : V \to C(K_2)$ is a nonlinear continuous operator. Then, for any $\varepsilon > 0$, there exist positive integers m, p, continuous vector functions $\mathbf{g} : \mathbb{R}^m \to \mathbb{R}^p$, $\mathbf{f} : \mathbb{R}^d \to \mathbb{R}^p$, and $x_1, x_2, \ldots, x_m \in K_1$ such that*

$$\left| \mathscr{G}(\mathbf{x})(\zeta) - \langle \underbrace{\mathbf{g}(\mathbf{x}(x_1), \mathbf{x}(x_2), \ldots, \mathbf{x}(x_m))}_{branch}, \underbrace{\mathbf{f}(\zeta)}_{trunk} \rangle \right| < \varepsilon$$

holds for all $\mathbf{x} \in V$ and $\zeta \in K_2$, where $\langle \cdot, \cdot \rangle$ denotes the dot product in $\mathbb{R}^p$. For the two functions $\mathbf{g}, \mathbf{f}$ classical deep neural network models and architectures can be chosen that satisfy the universal approximation theorem of functions, such as fully-connected networks or convolutional neural networks.

The interested reader can find more information and details regarding the proof of the theorem in[5].

Fourier neural operator

The backbone algorithm of the Fourier neural operator (FNO) was originally introduced with the kernel integral operators in[4], while the actual model was proposed in[3] and is based on the idea of parameterizing the integral kernel in the Fourier space. Similarly to DeepONet, FNO aims to learn a mapping between two infinite dimensional (functional) spaces. The method employs an iterative algorithm to predict a sequence of functions $v_0 \mapsto v_1 \mapsto \cdots \mapsto v_T$ taking values in $\mathbb{R}^{d_v}$ formally defined as

$$v_{t+1}(x) := \sigma\Big(W v_t(x) + (\mathscr{K}(\mathbf{x};\phi) v_t)(x)\Big), \quad \forall x \in \Omega \tag{2}$$

where $\mathscr{K} : \mathscr{X} \times \Theta_{\mathscr{K}} \to \mathscr{H}(\mathscr{Y}(\Omega;\mathbb{R}^{d_v}), \mathscr{Y}(\Omega;\mathbb{R}^{d_v}))$ maps to bounded linear operators on $\mathscr{Y}(\Omega;\mathbb{R}^{d_v})$ and is parameterized by $\phi \in \Theta_{\mathscr{K}}, W : \mathbb{R}^{d_v} \to \mathbb{R}^{d_v}$ is a linear transformation and $\sigma : \mathbb{R} \to \mathbb{R}$ is an activation function to introduce non-linearity. The kernel integral operator $\mathscr{K}(\mathbf{x};\phi)$ is defined as

$$\big(\mathscr{K}(\mathbf{x};\phi) v_t\big)(x) := \int_{\Omega} \kappa\big(x, y, \mathbf{x}(x), \mathbf{x}(y); \phi\big) v_t(y) dy, \quad \forall x \in \Omega \tag{3}$$

where $\kappa_\phi : \mathbb{R}^{2(d+d_x)} \to \mathbb{R}^{d_v \times d_v}$ is approximated by a neural network parameterized by $\phi \in \Theta_{\mathscr{K}}$. In FNO, the kernel integral operator in Eq. 3 is replaced with a convolution operator defined in Fourier space. The dependence on the input function $\mathbf{x}$ is removed by imposing $\kappa_\phi(x,y) = \kappa_\phi(x-y)$ and thus the operator in Eq. 3 results in

$$\big(\mathscr{K}(\mathbf{x};\phi) v_t\big)(x) = \mathscr{F}^{-1}\Big(\mathscr{F}(\kappa_\phi) \cdot \mathscr{F}(v_t)\Big)(x), \quad \forall x \in \Omega \tag{4}$$

where $\mathscr{F}$, $\mathscr{F}^{-1}$ denote the forward and inverse Fourier transformation of a function $f : \Omega \to \mathbb{R}^{d_v}$ defined as

$$(\mathscr{F} f)_j(k) = \int_{\Omega} f_j(x) e^{-2i\pi\langle x,k \rangle} dx, \qquad (\mathscr{F}^{-1} f)_j(x) = \int_{\Omega} f_j(k) e^{2i\pi\langle x,k \rangle} dk, \tag{5}$$

where $k \in \Omega$ represents the frequency modes and $j = 1, \ldots, d_v$ with $i = \sqrt{-1}$ the imaginary unit. For implementation purposes a finite-dimensional parameterization is chosen by truncating the Fourier expansion with a maximal number of modes $k_{\max} = |Z_{k_{\max}}| = |\{k \in \mathbb{Z}^d : |k_j| \leq k_{\max,j}, \text{ for } j = 1, \ldots, d\}|$. The low frequency modes are chosen by defining an upper-bound on the ℓ_1-norm of $k \in \mathbb{Z}^d$.

The complete FNO algorithm is employed as follows. An input $\mathbf{x} \in \mathscr{X}$ is first lifted to a higher dimensional representation $v_0(x) = P(\mathbf{x}(x))$ parameterized by a shallow FNN. Subsequently, a number of iterations of updates are applied $v_t \mapsto v_{t+1}$ through

a series of Fourier layers. At each Fourier layer, and given that Ω is discretized with $m \in \mathbb{N}$ points we have that $v_t \in \mathbb{R}^{m\times d_v}$ and $\mathscr{F}(v_t) \in \mathbb{C}^{m\times d_v}$ which results to $\mathscr{F}(v_t) \in \mathbb{C}^{k_{\max}\times d_v}$ after the truncation of the higher order modes. In practice, it has been shown that $k_{\max,j} = 12$ perform satisfactorily for most applications. Next, the output is multiplied to a weight tensor $R \in \mathbb{C}^{k_{\max}\times d_v\times d_v}$. For a uniform discretization, $\mathscr{F}$ is replaced with a Fast Fourier Transform (FFT) which greatly reduces algorithmic complexity from $\mathscr{O}(m^2)$ to $\mathscr{O}(m\log m)$. After the inverse Fourier transform the output is added to another weight matrix which is multiplied with the input i.e., $Wv_t(x)$, and finally the result is passed through a non-linear activation function $\sigma(\cdot)$. After a series of T Fourier layers, the PDE output $\mathbf{y}(x) = Q(v_T(x))$ is computed via the transformation of v_T with $Q : \mathbb{R}^{d_v} \to \mathbb{R}^{d_y}$.

In the original work, two main FNO models are proposed: the **FNO-2D** and **FNO-3D**. In FNO-3D, 3-D convolutions are performed (in space and time) and the model maps 3D functions representing the initial time steps to 3D functions representing the full trajectory. It has been shown that FNO-3D is more expressive and leads to better accuracy for sufficient data. However, it is fixed to the training interval, so once trained, it can only predict the solution in this range but for any time-discretization. On the other hand, FNO-2D, performs 2-D convolutions together with a recurrent architecture to propagate in time. While the advantage of this approach is that the model can predict the solution for any number of time steps (and for fixed time interval Δt), it has been shown that it is less expressive and more challenging to train. For more information, the interested reader is referred to[3].

Data generation

Brittle fracture mechanics

In this application, we consider a continuum fracture modeling method (the second-order phase field model), to approximate the growth of fracture on a unit square plate, which is fixed on the bottom and the left edge, subjected to displacement controlled shear loading conditions on the top edge[2]. We specifically aim to approximate the mapping $\mathscr{G} : H(x,t=0;l_c,y_c) \mapsto \phi(x,t)$. We consider the material parameters as: $\lambda = 121.15$ kN/mm^2, $\mu = 80.77$ kN/mm^2 and $G_c = 2.7\times 10^{-3}$ kN/mm, where λ and μ are Lamé's constants. The computation is performed by applying constant displacement increments of $\Delta u = 1\times 10^{-4}$ mm to effectively capture the crack propagation. For all simulations, l_0 is considered to be 0.0125 mm.

Initial cracks are modeled by using the local strain-history function, $H(x,t)$. The initial strain-history function, $H(x,t=0)$ is defined as a function of the closest distance of any point, x, on the domain to the line, l, which represents the discrete crack[2]. In particular, it is set as:

$$H(x,t=0;l_c,y_c) = \begin{cases} \frac{BG_c}{2l_0}\left(1-\frac{2d(x,l)}{l_0}\right) & d(x,l) \leqslant \frac{l_0}{2} \\ 0 & d(x,l) > \frac{l_0}{2} \end{cases}, \tag{6}$$

where B is a scalar parameter that controls the magnitude of the scalar history field and for this experiment is considered as $B = 10^3$ based on domain knowledge. The function $d(x,l)$ computes the distance between the middle horizontal line (defined by the two parameters l_c, y_c) of the crack and sets the appropriate value for the initial strain functional. The simulation takes place in a rectangular domain $\Omega = [0,1]\times[0,1]$, discretized with $m_s \times m_s = 162\times 162$ mesh points. The quasi-static problem is solved and in total $m_t = 8$ snapshots of the phase field $\phi(x)$ are considered. Thus the dimensionality of input and output realizations is $D_x = 26,244$ and $D_y = 209,952$ respectively. In total, we generate $N = 261$ data and split to $N_{\text{train}} = 230, N_{\text{test}} = 31$ for testing and training respectively. Figure 1 depicts the simulation box with the associated varying parameters as well as a representative realization of the model with the propagation of an initial crack through the phase field quantity in three points in time. The training datasets are generated using the code developed in[?], which is available on `https://github.com/somdattagoswami/IGAPack-PhaseField`.

Rayleigh-Bénard fluid flow convection

In this problem, we aim to approximate the operator $\mathscr{G} : T(x,t=0) \mapsto T(x,t)$, which maps the initial temperature field to its entire time evolution. The simulation takes place in a spherical domain $\Omega = [0,4]\times[0,1]$, discretized with $m_s \times m_s = 128\times 128$ mesh points. For each realization, the PDE is solved in the time interval $t = [0,1]$ for $\delta t = 10^{-2}$ and $m_t = 40$ times steps are considered from the 100. The dimensionless Rayleigh number is set equal to $2\cdot 10^6$, while the Prandtl number is set equal to 1. Thus the dimensionality of input and output realizations are $D_x = 16,384$ and $D_y = 655,360$ respectively. In total, we generate $N = 800$ data and split to $N_{\text{train}} = 720, N_{\text{test}} = 80$ for testing and training respectively. In Figure 2, a schematic of the convective flow and a random realization of the evolution of the temperature field $T(x,t)$ are shown. Datasets were generated using the *Dedalus Project* that can be found in `https://github.com/DedalusProject/dedalus`.

Shallow-water equations

In this problem, we aim to approximate the operator between the random Gaussian perturbation h' to the time-evolved velocity component u, i.e., $\mathscr{G} : h'(\lambda,\phi,t=0) \mapsto u(\phi,\lambda,t)$. The constants are defined as: $\Xi = 7.292\times 10^{-5}$ s^{-1} is the Earth's angular

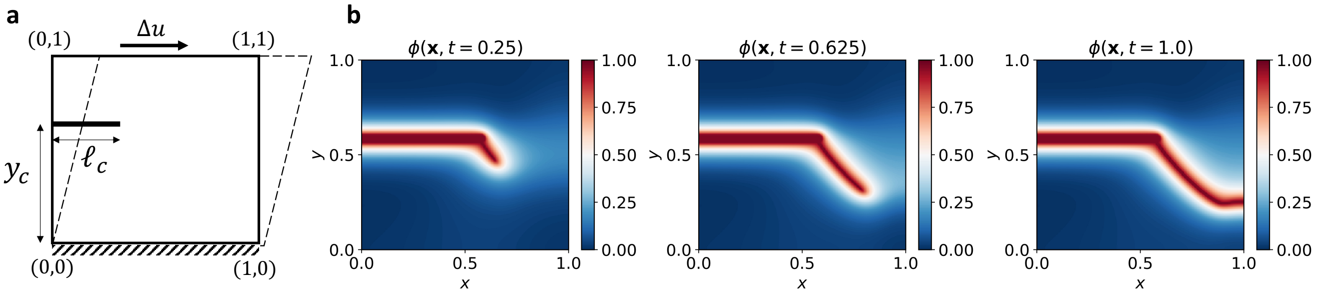

Supplementary Figure 1. (a) Schematic of the simulation box considered in the generating labeled dataset for brittle fracture under shear loading, depicting the two random parameters, namely the length of the crack (l_c) and the height of the crack (y_c) and (b) resulting phase field $\phi(x)$ from the solution of the PDE model, showing the evolution of the crack through three-time steps $t = \{0.25, 0.625, 1\}$.

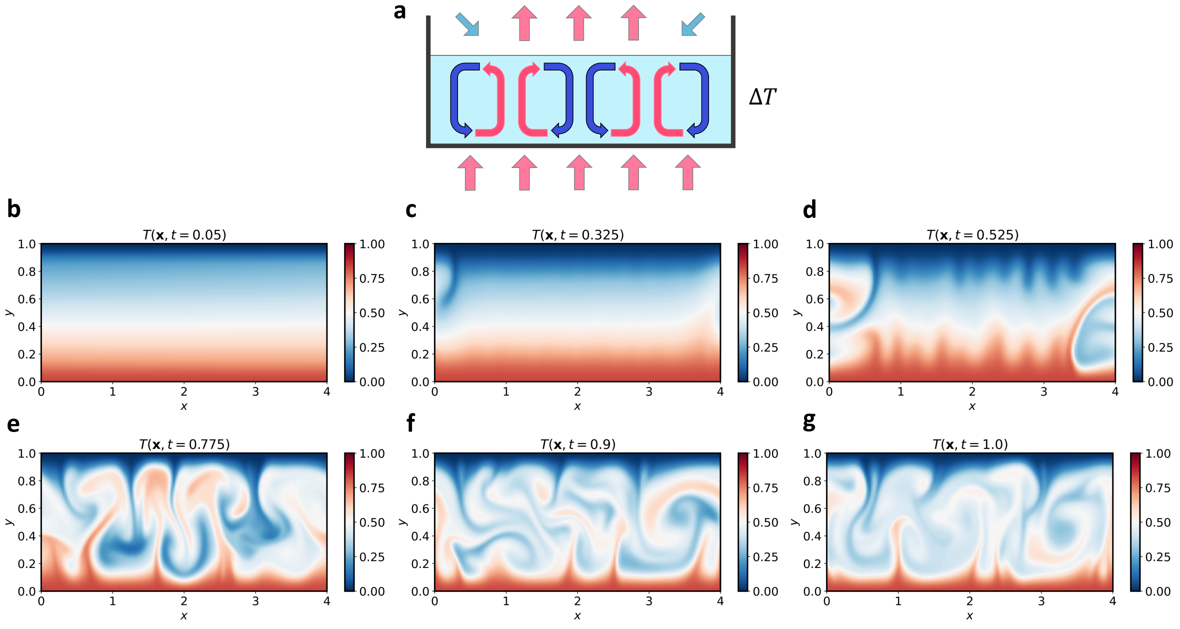

Supplementary Figure 2. (a) Schematic of the Rayleigh-Bénard convective flow in a thin fluid layer due to temperature gradient ΔT with the creation of convective cells at the top and (b)-(g) the evolution of the temperature field $T(x,t)$ for a random realization of the initial temperature field for six time steps $t = \{0.05, 0.325, 0.525, 0.775, 0.9, 1.0\}$ based on the numerical solution of the PDE.

velocity, $g = 9.80616$ ms^{-1} the gravitational acceleration, $\nu = 1.0 \times 10^5$ m^{2}s^{-1} the diffusion coefficient, $u_{\max} = 80$ ms^{-1}, $\phi_0 = \pi/7$, $\phi_1 = \pi/2 - \phi_0$, thus the mid-point of the jet where the maximum velocity is applied is at $\phi = \pi/4$. The initial velocity u is defined, so that it is zero outside the zone of interest with no discontinuities in the northern and southern poles. The parameters of the Gaussian perturbation which is added to the height field are set as: $\phi_2 = \pi/4$, $\hat{h} = 120$ m, while α, β are random parameters. In this expression, the Gaussian functions are multiplied with a cosine so that the forced perturbation is zero at the two poles.

While the initial condition of the velocity field is given analytically (see Main Text), the height field is obtained by numerically integrating the balance equation

$$gh(\phi) = gh_0 - \int^{\phi} \alpha u(\phi') \left[f + \frac{\tan(\phi')}{\alpha} u(\phi') \right] d\phi', \tag{7}$$

where $\alpha = 6.37122 \times 10^6$ m is the radius of the Earth and h_0 is set so that mean layer depth around the sphere is equal to 10 km. The above integral can be calculated using a numerical scheme such a Gaussian quadrature. The Gaussian perturbation $h'(\lambda, \phi, t = 0)$, is added to the initial height field computed by the expression above to form the final initial condition $h(\lambda, \phi, t = 0)$.

The simulation takes place in a spherical domain $\Omega = [-\pi, \pi] \times [-\pi, \pi]$, discretized with $m_s \times m_s = 256 \times 256$ mesh points in the longitudinal and latitudinal direction respectively. The PDE is solved in the time interval $t = [0, 360h]$ for $\delta t = 1.\bar{6} \cdot 10^{-1} h$ and in total $m_t = 72$ times steps (equispaced) are considered. For the presentation of results, the time range is mapped to the dimensionless range $t = [0, 1]$. Thus the dimensionality of input and output realizations is $D_x = 65,536$ and $D_y = 4,587,520$ respectively. The significantly high dimensionality of outputs makes this problem particularly challenging. In total, we generate $N = 300$ data and split to $N_{\text{train}} = 260, N_{\text{test}} = 40$ for testing and training respectively. The evolution of the velocity field u for a random realization of the initial height field is shown in Figure 3 for six points in time. Datasets were generated using the *Dedalus Project* that can be found in https://github.com/DedalusProject/dedalus.

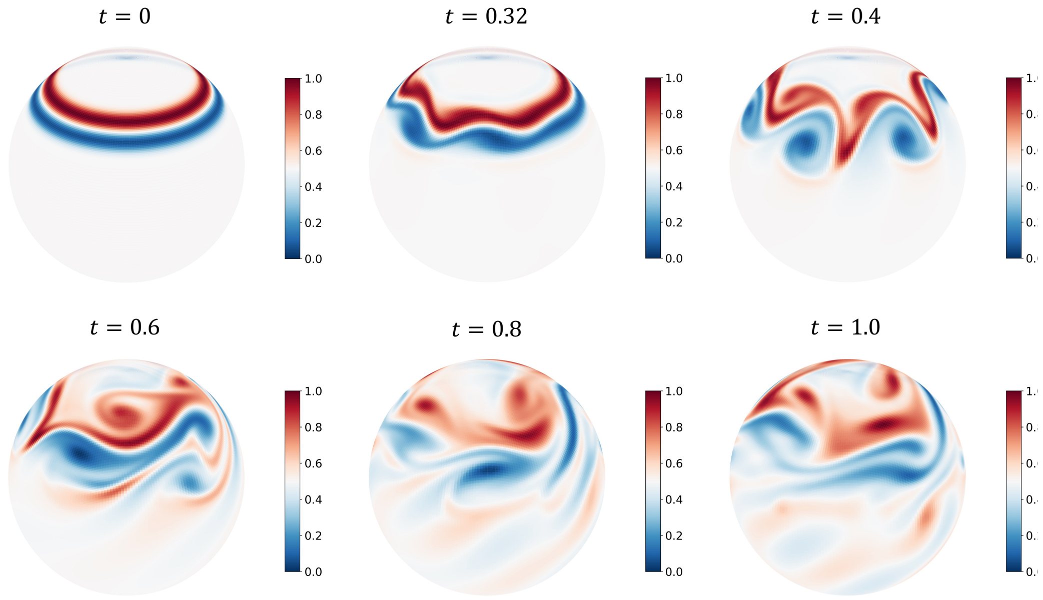

Supplementary Figure 3. Evolution of the velocity field $u(\lambda, \phi)$ on a sphere (Earth) as a solution of the spherical shallow-water equations, for a random realization of the initial perturbation to the height field, i.e., $\alpha = 0.38, \beta = 0.20$. The velocity field is shown for six time steps $t = \{0, 0.32, 0.4, 0.6, 0.8, 1.0\}$.

Network architecture details

The proposed approach employs autoencoders to reduce the dimensionality of input and output PDE data and feed the DeepONet model. Although the framework is general enough and any suitable autoencoder model can be used, including convolutional autoencoders (CAE) and variational autoencoders (VAE), we found that simple multi-layer autoencoders (MLAE) resulted in the best L-DeepONet performance. Due to the large number of available training data (each output snapshot is considered a training image), all autoencoders result in very good reconstruction accuracy. However, not all autoencoders construct a latent space which is suitable for the training of DeepONet. The choice of the autoencoder also depends on the choice of the DeepONet architecture. For example, if a CNN is employed in the DeepONet's branch net, then it is not advised to use CAE for dimension reduction as the input functions will be convolved twice.

Tables 2, and 3, show the architecture of the autoencoders and the neural operators. For all trained multi-layer autoencoders the depth and width are chosen based on the dimensionality of the original data. For the neural operators, a standard architecture is chosen which resulted in a good performance for all applications. Finally, for training both FNO-2D and FNO-3D the code from the original implementation was used which can be found at https://github.com/zongyi-li/fourier_neural_operator.

Supplementary Table 2. Architecture of multi-layer autoencoders (MLAE). Parameter d represents the dimensionality of the latent space. All layers use the ReLU activation function except the last one which uses the Sigmoid function.

Application	MLAE
Brittle material fracture	$[128, 64, d, 64, 128]$
Rayleigh-Bénard fluid flow	$[400, 256, 169, d, 169, 256, 400]$
Shallow water equation	$[256, 169, 121, d, 121, 169, 256]$

Supplementary Table 3. Architecture of DeepONet. Inputs to the Conv2D layers consist of the number of output filters, kernel size, and activation function respectively. Parameter p has been set equal to 5.

Branch net	Trunk net
Conv2D(32, (3,3), sine)<br>Batch normalization<br>Conv2D(16, (3,3), sine)<br>Batch normalization<br>Conv2D(16, (3,3), sine)<br>Batch normalization<br>Dense($d * p$)	Dense(100)<br>Activation(sine)<br>Dense(100)<br>Activation(sine)<br>Dense($d * p$)

Supplementary Results

Error plots

In Figures 4, 5, and 6, the error plots corresponding to the three applications for all studied models are presented for a single random realization. The error fields represent the point-wise absolute error between the reference response and model prediction. As shown and discussed in the main paper, L-DeepONet results in the smallest interpolation error across diverse applications.

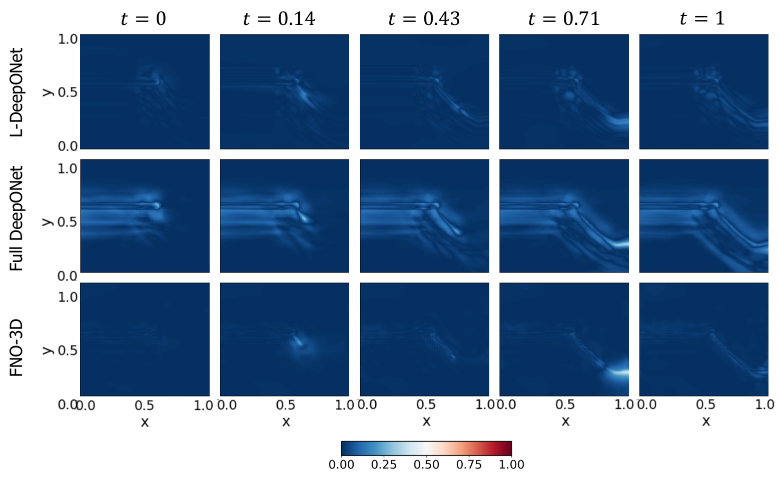

Supplementary Figure 4. Brittle fracture in a plate loaded in shear: absolute error plots of all the neural operators for the results of the representative sample with $y_c = 0.55$ and $l_c = 0.6$ shown in Fig. 3. The neural operator is trained to approximate the growth of the crack for five time steps from a given initial location of the defect on a unit square domain.

Results using principal component analysis (PCA)

In Figure 7, we provide the results of the PCA-based L-DeepONet. In this scenario, the PCA is performed on the combined dataset of both input and output data. The left plots in Figure 7, show the reconstruction MSE of the PCA method for all three PDE applications, whereas the plots on the right show the MSE of the neural operators. First, we observe that for certain problems the PCA results in low predictive accuracy for very small values of the latent dimensionality ($d = 9$ in Figure 7 a, and

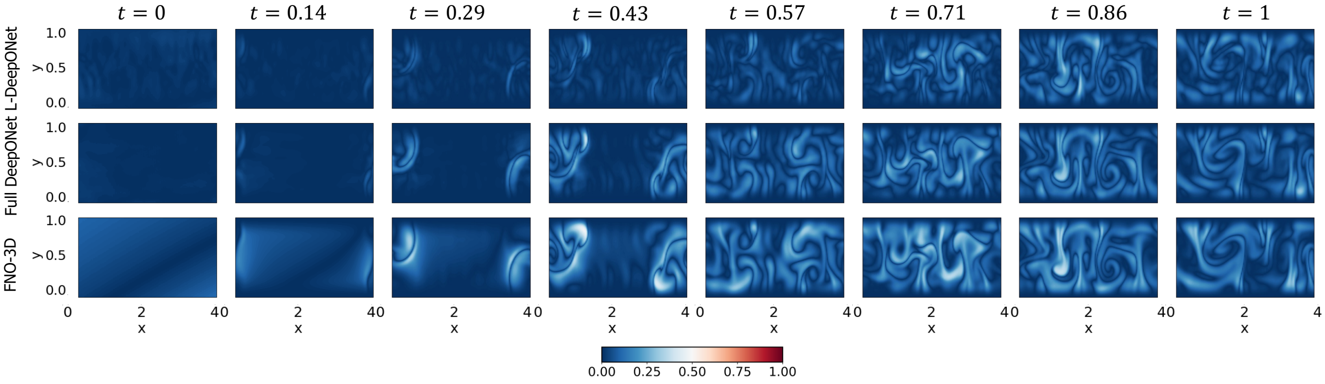

Supplementary Figure 5. Rayleigh-Bénard convective flow:

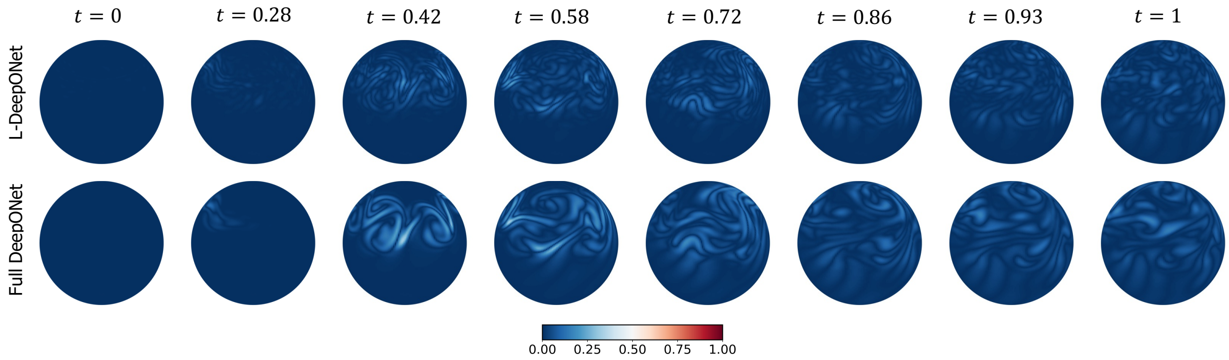

Supplementary Figure 6. Shallow water equations: absolute error plots of the predictions of the temperature field from a given initial temperature as obtained for all the neural operators. The predicted solution is shown in Fig. 4.

$d = 25$ in Figure 7 c). This result is also reflected in the low predictive accuracy of the neural operator model. In Figure 7 b, we observe that the performance of PCA and L-DeepONet when compared with the autoencoder results in the main text (Figure 2), is comparable. However, for the third and most challenging problem we found that the autoencoder (see Figure 2 in main text) outperforms the PCA-based L-DeepONet for all tested values of d (see Figure 7 c). To summarize, we found that the autoencoder-based L-DeepONet results in a better overall performance (especially for low d) with an accuracy that is either comparable or better to the PCA-based L-DeepONet. However, in certain problems PCA can performance as good as the AE, with the additional advantage of being much less computationally expensive.

Results using U-shaped Neural Operator (U-NO)

The U-shaped neural operator (U-NO)[6] architecture mirrors the U-Net design within FNO, enabling the efficient training of overparameterized models and harnessing the advantages of deep neural networks. Adhering to the U-shaped architecture paradigm, the U-NO employs a two-fold process. Initially, it systematically maps the input function to functions characterized by smaller domains, a phase referred to as encoding. Subsequently, it reverses this operation during decoding, generating a fitting output function. Notably, skip connections from the encoder component enhance the efficiency and effectiveness of this process. Akin to FNO, U-NO employs convolutional kernel operations to approximate the linear integral operation in the Fourier domain using the fast Fourier transform.

While the U-Net framework in U-NO is often divided into encoder and decoder segments, it's important to note that these segments don't operate in the conventional sense of encoder and decoder components found in autoencoder architectures. In autoencoder setups, a singular latent space (L) is established, involving a non-linear transformation from input (X) to this space ($E : X \to L$), along with a corresponding mapping from the latent space to the output ($D : L \to Y$). The encoder modifies the representation of each sample within the latent space, and the decoder reconstructs outputs based solely on this modified

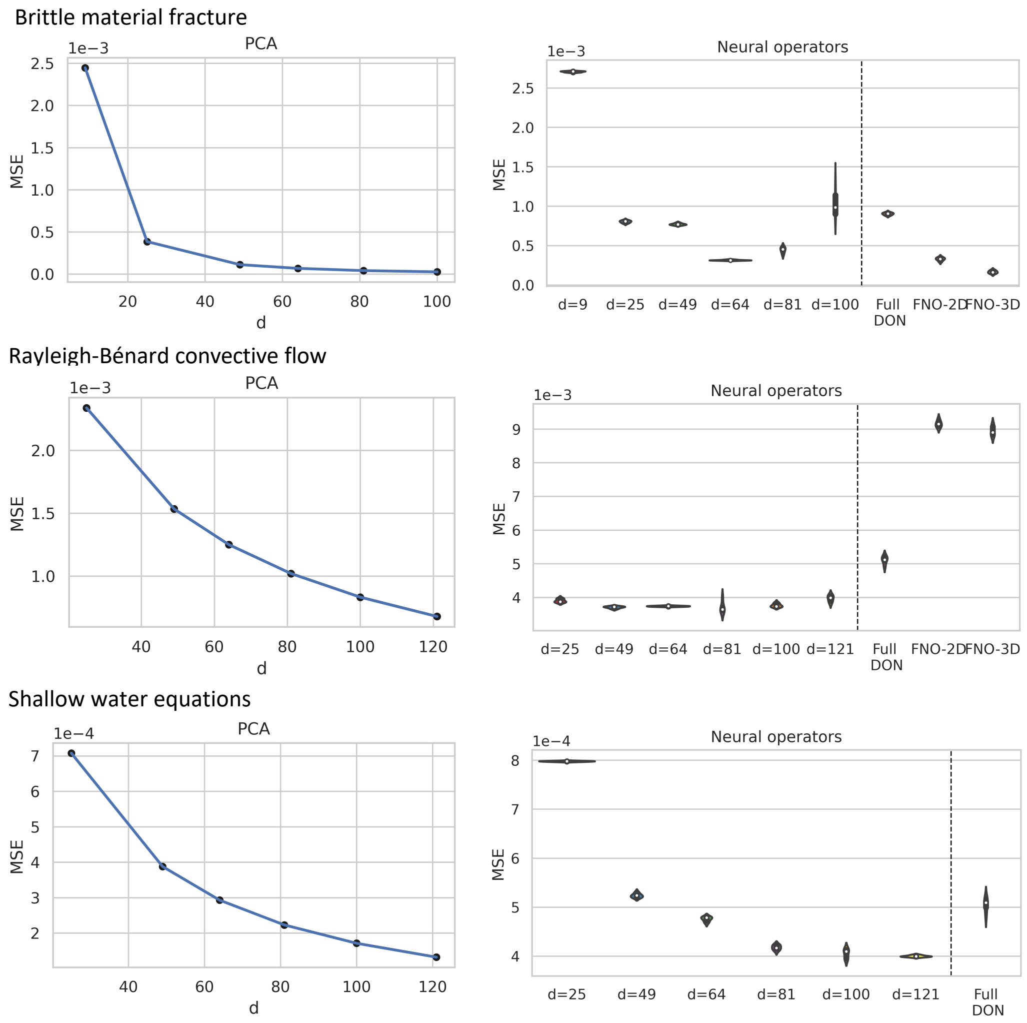

Supplementary Figure 7. Results for all applications of principal component analysis (PCA) (left plots) for different values of the latent dimensionality and neural operators (right plot) for all studied models. Violin plots represent 5 independent trainings of the models using different random seed numbers.

representation. This structure allows for the separation of the encoder and decoder components, enabling their independent use[7]. In contrast, in a U-NO architecture, the output mapping is directly connected to the input space. Both the input and all intermediate representations are crucial for generating the output. Consequently, the encoder and decoder in a U-NO cannot be treated as two independent entities for computing the output. Moreover, the U-NO architecture provides a discrete basis as opposed to DeepONet, which has a continuous trunk network basis.

In Table 4, we have presented the results of UNO for all the applications considered in this work. It is crucial to highlight that U-NO encounters the same computational challenges as FNO in the context of high-dimensional PDEs due to the intensive computational demands associated with the Fourier transform. Therefore, for the Shallow water equation, the computational time and accuracy could not be reported.

Supplementary References

1. Chen, T. and Chen, H. Universal approximation to nonlinear operators by neural networks with arbitrary activation functions and its application to dynamical systems. IEEE Transactions on Neural Networks, **6**, 911–917 (1995).

2. Goswami, S. Phase field modeling of fracture with isogeometric analysis and machine learning methods (Doctoral Thesis, 2021).

Supplementary Table 4. Accuracy, computational time in seconds and number of trainable parameters for U-NO across all considered applications.

Application	Accuracy	Time/epoch	# trainable parameters
Brittle material fracture	$0.16 \cdot 10^{-4}$	3,136	1,280,000
Rayleigh-Bénard fluid flow	$2.22 \cdot 10^{-4}$	7,763	70,993,525
Shallow water equation	$--$	$--$	1,135,887,697

3. Li, Z., Kovachki, N., Azizzadenesheli, K., Liu, B., Bhattacharya, K., Stuart, A. & Anandkumar, A. Fourier Neural Operator for Parametric Partial Differential Equations. Preprint at https://arxiv.org/abs/2010.08895 (2020).

4. Li, Z., Kovachki, N., Azizzadenesheli, K., Liu, B., Bhattacharya, K., Stuart, A. & Anandkumar, A. Neural operator: Graph kernel network for partial differential equations. Preprint at https://arxiv.org/abs/2003.03485 (2020).

5. Lu, L., Jin, P., Pang, G., Zhang, Z. & Karniadakis, G. E. Learning nonlinear operators via DeepONet based on the universal approximation theorem of operators. Nature Machine Intelligence, **3**, 218–229 (2021).

6. Rahman, M. A., Ross, Z. E. & Azizzadenesheli, K. U-NO: U-shaped Neural Operators. Preprint at https://arxiv.org/abs/2204.11127 (2022).

7. Schlegl, T., Seeböck, P., Waldstein, S. M., Langs, G., & Schmidt-Erfurth, U. f-AnoGAN: Fast unsupervised anomaly detection with generative adversarial networks. Medical image analysis, **54**, 30-44 (2019).
